# Early abnormal transient hyperemic response test can predict delayed ischemic neurologic deficit in subarachnoid hemorrhage

**DOI:** 10.1186/s13089-017-0079-7

**Published:** 2018-01-04

**Authors:** Hosam Al-Jehani, Mark Angle, Judith Marcoux, Jeanne Teitelbaum

**Affiliations:** 1 Neurocritical Care Unit, Montreal Neurological Institute and Hospital, McGill University, Montreal, QC Canada; 2 Department of Neurosurgery and Critical Care Medicine, King Fahad University Hospital, Imam Abdulrahman Bin Faisal University, Dammam, Saudi Arabia; 3 Department of Neurology and Neurosur- gery, Montreal Neurological Institute and Hospital, Montreal General Hospital, McGill University, Montreal, QC Canada; 4 Department of Neurosurgery, King Fahad Specialist Hospital in Dammam, Dammam, Saudi Arabia

**Keywords:** Transient hyperemic response test (THRT), Delayed ischemic neurologic deficit (DIND), Vasospasm (VS), Subarachnoid hemorrhage (SAH)

## Abstract

**Background:**

Early detection of vasospasm is crucial to prevent significant delayed ischemic neurological deficit post subarachnoid hemorrhage. The standard methods of detection, including cerebral angiogram and computed tomography are invasive and not safe to be repeated, as is very often indicated clinically. Transient hyperemic response test has been previously used to predict autoregulation failure in traumatic brain injury and subarachnoid hemorrhage.

**Aims:**

We investigate the usability of transient hyperemic response test as a predictor of clinical vasospasm in a cohort of patients with aneurismal subarachnoid hemorrhage.

**Methods:**

A retrospective review of all THRT examinations done between January 2011 and July 2012 conducted at Montreal Neurological Institute and Hospital and the Montreal General Hospital. Patients diagnosed with aSAH in which the THRT was performed within the first 24–48 h of admission were included in the study. Two-dimensional transcranial Doppler images were obtained and velocities were recorded. A positive response was one in which the velocity was increased by more than 9% of the baseline systolic velocity, indicating an intact cerebral autoregulation. Lindegaard ratio > 3 is considered abnormal and in the context of elevated systolic velocity of the MCA, is highly suggestive of DIND.

**Results:**

Fifteen patients met the inclusion criteria. A total of 6 patients developed clinical and radiological vasospasm. Out of these 6 patients, 5 (83%) had an abnormal THRT in the initial TCD assessment (*p* = 0.0406). We found that abnormal transient hyperemic response test readings are predictive of subsequent vasospasm development.

**Conclusions:**

The results of this small retrospective study support the notion that transient hyperemic response test has predictive value in vasospasm development and may prove useful in patient monitoring and successful clinical management.

## Background

Vasospasm (VS) and its clinical counterpart, delayed ischemic neurological deficit (DIND), are common causes of morbidity and mortality in patients suffering from subarachnoid hemorrhage (SAH). There are multiple causes of VS but the most frequent are traumatic brain injury and aneurysmal SAH [1, 2]. Aneurysmal SAH carries a significant risk of morbidity and high mortality rate. Survivors of SAH are often faced with sequelae of their hemorrhage and as many as 17–40% of SAH sufferers develop vasospasm and subsequent neurological deficits [1, 2]. As a result, only a small percentage of all survivors return to their premorbid “normal” condition.

DIND is a common complication clinically detected in 30–40% of patients with aneurysmal SAH, although it might be angiographically present in up to 70% of a SAH patients [3, 4]. It usually begins between day 4 and 7 post aneurysmal SAH episode and is heralded by a new neurological deficit or worsening of an existing one. Other metabolic and homeostatic derangements such as inflammation, microvascular dysfunction, or cerebral edema have also been described [3, 5].

Although the gold standard for diagnosis of vasospasm and DIND is a cerebral angiogram [6], several non-invasive tests are available to clinicians to better detect vasospasm. These include, but not limited to TCD, [7, 8] and more recently improved application in CT perfusion [9, 10]. However, for the most part, these applications are non-diagnostic of DIND. Another major concern with these imaging modalities is that irrespective of how informative they are, the radiation doses and contrast material involved in their generation, limit their utility as dynamic tests to go along with the clinical evolution in the short-term or better still, in real time. This has major drawbacks for the very nature of the decision-making process required in a critical care setting. This raises the need for a test that can be safely repeated and is informative in the realm of parameters affecting clinical care decisions.

Here, we investigate the utility of THRT as a predictor of clinical DIND in a cohort of patients with aSAH. The results of this small retrospective study may shed some light on the initial autoregulatory mechanism in patients presenting with aSAH.

## Methods

### Study design and equipment

A retrospective review of all THRT examinations done between January 2011 and July 2012 conducted at the intensive care units of the Montreal Neurological Institute and Hospital and the Montreal General Hospital. Both of these hospitals are tertiary care neurology and neurosurgery centers. All the procedures were carried out after ethical review and according to the hospitals safety protocols. Patient consent was received prior to accessing retrospective data. All of the TCD examinations were performed by a single operator (HA). All examinations were performed on a Zonare TCD ultrasound machine using the modifiable range 2–4 m Hz P4-1c Phased Array probe (ZONARE Medical Systems, Inc., Mountain View, CA, USA).

### Sample selection

Twenty-one patients with a diagnosis of aSAH in which the THRT was performed within the first 24–48 h of admission were included in the study. Patients for whom the TCD examination was not possible, due to absent or suboptimal temporal insonation window, were excluded. Patients unable to tolerate the THRT due to induced bradycardia were also excluded. To harmonize the cohort, patients with severe carotid artery disease as well as with traumatic SAH associated with significant other traumatic intracranial lesions, such as contusions and diffuse brain swelling, were excluded from the sample.

### Data acquisition

Two-dimensional transcranial Doppler images were obtained and velocities were recorded. The THRT technique was used according to the method described previously [11]. In brief, while insonating the middle cerebral artery (MCA), the common carotid artery was occluded in the neck. The carotid artery was identified either by circulation arrest on the Doppler signal of the MCA or, to account for cross flow from the contributors of circle of Willis, more than 30% reduction of the systolic velocity of the middle cerebral artery. The compression was carried out for 5–9 s. Then, upon restoration of circulation, the first systolic peak was ignored and the following three systolic velocity peaks were kept for analysis and averaged. A positive response was one in which the velocity was increased by more than 9% of the baseline systolic velocity, indicating an intact cerebral autoregulation. The TCD was repeated every 48 h.

### Data analysis

Lindegaard ratio is defined as the ratio between the velocities in the MCA to that of the extracranial internal carotid artery. A ratio greater than three is considered abnormal, and in the context of elevated systolic velocity of the MCA, is highly suggestive of DIND. In some patients, a computerized tomography angiogram or a formal digital subtraction angiography was performed within the first 24–48 h of admission and this data was included in the analysis of our cohort. The statistical analysis of calculating the means, Fisher contingency tables, and the likelihood ratio were performed on Graph pad prism software (version 4.0, 2010).

## Results

### Patient data, demographics, and incidence of DIND

A total of 21 patients were initially reviewed. Three patients with traumatic SAH due to associated contusions and diffuse brain swelling, and two other patients with carotid artery disease in the form of fibromuscular dysplasia in one and severe atherosclerotic disease in the other were excluded. Two patients suffered a transient bradycardic response at the time of the first carotid compression and further testing was abandoned with no other adverse events reported due to the administration of the test. The remaining 15 patients were: 11 females and four males with an age ranging from 42 to 74 years with resulting sample mean age of 54.8 years. The distribution of these patients along the Hunt and Hess scale, location of aneurysms, and treatment offered to secure the aneurysms are presented in Table 1. The majority of the patients were grade 1–3 (*n* = 10) on the Hunt and Hess scale and the remainders were grade 4–5 (*n* = 5). Five patients were submitted to microsurgical clipping of the aneurysms and eight for endovascular coiling. Two patients were considered idiopathic SAH as no source of the SAH was identified on vascular imaging. Of the 15 patients treated in the NICU, six (40%) developed symptomatic DIND necessitating introduction of the Montreal Neurological Hospital’s protocol. Two additional patients developed an asymptomatic vasospasm seen on vascular imaging with no treatment offered giving rise to a total incidence in vasospasm of 53.3%.

**Table 1:** Patient demographics

Patient no.	Age and gender	Location of aneurysms	Hunt and Hess grade	Treatment
1	53, F	SAH, MCA	1	Clipping
2	54, F	SAH, MCA, ICH	4	Clipping
3	57, M	SAH, Acom	2	Coiling
4	49, M	SAH, MCA, ICH	5	Clipping
5	48, F	SAH, MCA	1	Coiling
6	45, F	SAH, negative	1	None
7	58, M	SAH, Pcom	1	Coiling
8	42, F	SAH, ICA, HIE	4	Coiling
9	49, F	SAH, negative	3	None
10	74, F	SAH, Pcom	1	Coiling
11	55, F	SAH, Acom	3	Clipping
12	48, F	SAH, Basilar, IVH	4	Coiling
13	74, F	SAH, Pcom	1	Coiling
14	43, F	SAH, MCA	3	Clipping
15	73, M	SAH, Pcom, IVH	2	Coiling

The patients' age, gender, location of aneurysm, distribution along the Hunt and Hess scale and treatment performed to secure the aneurysm of the fifteen patients taking part on the study

*SAH* subarachnoid hemorrhage, *MCA* middle cerebral artery, *ICH* intracerebral hemorrhage, *Acom* anterior communicating artery, *Pcom* posterior communicating artery, *HIE* hypoxic ischemic encephalopathy, *IVH* intraventricular hemorrhage

### Early abnormal THRT predicted development of DIND

On initial TCD evaluations, five patients had MCA velocity greater than 120 cm/s. Of those patients, only one patient developed vasospasm in subsequent course. In comparing the group of patients with symptomatic vasospasm (*n* = 6) to those with asymptomatic or no vasospasm (*n* = 9), we found that five out of the six patients (83%) in the vasospasm group had an abnormal THRT (*p* = 0.0406, Fisher’s exact test, two-tailed) (sensitivity 83%, specificity 78%, PPV 71%, NPP 88%) (Table 2).

**Table 2:** Abnormal THRT is predictive of subsequent vasospasm development

	Vasospasm	Asymptomatic or no vasospasm	Total
Abnormal THRP	5	2	7
Normal THRP	1	7	8
Total	6	9	15

As many as 83% patients who developed vasospasm had an abnormal THRP (*p* = 0.0406, Fisher’s exact test, *n* = 15)

Two patients with asymptomatic vasospasm showed an abnormal THRT, and with the global cohort incidence of vasospasm of 53.3% (8 out of 15 patients); there is a significant predictability of THRT to future development of vasospasm (*p* = 0.0002, Fisher’s exact test, two-tailed, likelihood ratio 3.4) (Table 3). This is comparable to the predictability of the Lindegaard ratio for vasospasm in our cohort (50% with vasospasm and 22% with no vasospasm, *p* = 0.0003, *data not shown*).

**Table 3:** Abnormal THRT can predict asymptomatic vasospasm development

	Vasospasm	No vasospasm	Total
Abnormal THRP	8	0	8
Normal THRP	0	7	7
Total	8	7	15

All patients including those who developed an asymptomatic vasospasm had an abnormal THRP prior to the event (*p* = 0.0002, Fisher’s exact test, *n* = 15)

### Radiological imaging is moderately predictive of DIND

In terms of radiological imaging, six patients had digital subtraction angiography (DSA), seven had computed tomography angiography (CTA), and computed tomography perfusion (CTP). Of the six patients with DSA, only two (33%) showed initial evidence of spasm and subsequently developed clinical DIND, with a similar percentage finding in the CTA group (*data not shown*).

When considering the CTP analysis, in the group with no vasospasm, none of the three patients showed any abnormality in the CTP maps including cerebral blood volume (CBV), cerebral blood flow (CBF), mean transit time (MTT), and time to peak (TTP). Of the remaining four patients who developed vasospasm, none of the patient showed abnormality neither in the CBV nor the CBF; while three patients (75%) showed prolongation of the MTT and TTP (*p* = 0.14, Fisher’s exact test, two-tailed) (Table 4).

**Table 4:** CTP analysis is moderately predictive of subsequent vasospasm development

	No vasospasm	Vasospasm	Total
Abnormal maps	0	3	3
Normal maps	3	1	4
Total	3	4	7

Only MTT and TTP maps in the CTP measurements showed abnormal measurements in the patients who developed vasospasm (*p* = 0.14, Fisher’s exact test, *n* = 7)

## Discussion

To prevent DIND related cerebral damage, it is recommended to commence a decisive treatment within 2 h of the onset of the symptoms [12]. This proves difficult to achieve in a clinical setting; firstly, due to the subtlety of the initial manifestation of DIND symptoms; and secondly, due to the difficulty of detecting a worsening neurological function in the presence of a major primary deficit. Therefore, this might not be a realistic endeavor in a lot of healthcare systems leaving many patients at a heightened risk of permanent deficits [2, 4].

The gold standard for the diagnosis of DIND is a cerebral angiogram [6]. It is an invasive procedure with inherent risk, including injury of the access artery and thromboembolic ischemic stroke [13]. Transcranial Doppler applications (TCD) have been used frequently as a non-invasive diagnostic method of DIND. The measurements are based on trends of increased systolic velocities and increased Lindegaard ratio (mean velocity in the middle cerebral artery/mean velocity in ipsilateral extracranial internal carotid artery ratio (MCA/ICA) to improve the specificity of diagnosis) [7, 8]. TCDs are a popular method because of their non-invasive nature and the fact that they do not have the side effects associated with the use of contrast agents and radiation. Moreover, they are easily accessible and pose no risk to the patient in terms of transportation while critically ill [7, 8].

Another method frequently used in the clinics is computed tomography perfusion (CTP) [9, 10]. Improvement in the imaging capabilities of CTP does allow for a non-invasive assessment of the cerebral circulation dynamics, but it is liable to the inter-evaluator variability and lack of clear interpretation paradigms given the multiplicity of variables involved in generating and influencing these images [9, 10]. To add to the complexity of this issue, we frequently find areas of clinical deterioration that do not match the areas of vascular spasm detected on vascular imaging, a phenomenon called “clinico-radiological dissociation”. Another early detection method is continuous electroencephalography (cEEG); able to detect changes suggestive of vasospasm [14]. However, for the most part, these applications are non-diagnostic of DIND. Another major concern with these imaging modalities is that irrespective of how informative they are, the radiation doses and contrast material involved in their generation limit their utility as dynamic tests to go along with the clinical evolution in the short-term or better still, in real time. This has major drawbacks for the very nature of the decision-making process required in a critical care setting. This raises the need for a test that can be safely repeated and is informative in the realm of parameters affecting clinical care decisions.

An invasive cerebral blood flow monitoring has the ability to detect changes conducive of vasospasm 24–48 h earlier than the onset of clinical vasospasm. This is suggestive of a failure of an initial autoregulatory mechanism that, if detected early, can be valuable in identifying patients at risk of developing DIND [15–18]. Transient hyperemic response test (THRT), also known as carotid compression test, is a type of TCD examination of the cerebral vessels. It was described for the first time by Giller in 1991 as a bedside test for the assessment of cerebral autoregulation with the basic premise that, in the presence of an intact autoregulation, the cerebral vessels will be able to contract in response to a hyperemic challenge generated by a brief carotid occlusion [11]. This would be translated in the TCD signal as an increase in systolic velocity. In a normal response, the velocity should increase by a minimum of 9% from the baseline velocity. Any value less than that or reduction of the velocity denoted a global failure of autoregulation. Since then, several studies have confirmed this concept in animal models, human subjects as well as several diseases including traumatic brain injury (TBI) and SAH [19–21] THRT has been used to predict autoregulation failure in traumatic brain injury as well as the development of autoregulation failure conducive of DIND in patients with SAH [20–22].

In our study, THRT was performed in the first 24–48 h and was abnormal in 83% of patient who subsequently developed DIND. Of interest, the two patients with radiological vasospasm also exhibited an abnormal THRT. This raises the possibility that the stress of SAH can prime the cerebral circulation into a deregulatory process that leads to the development of vasospasm, whether clinically overt or silent. Moreover, this predictability was not influenced by the clinical grade of the SAH, an observation that can lead to an unbiased and more attentive attitude toward patients at theoretical risk of DIND and establish lower threshold for their investigation and escalation of therapy when necessary. Although not reported in this study results, we occasionally detected the resolution of the autoregulation failure by the return of the normal response despite the aggressive therapy for vasospasm. This at least in theory can guide the timing of weaning of DIND treatment with an aim to reduce treatment morbidity.

Lam et al. reported the use of THRT in post-clipping patients performed 0–5 days post bleed, with five out of six patients exhibiting abnormal THRT [20]. Another study compared three autoregulatory parameters: THRT, Sxa (based on TCD), and TOxa (based on near-infrared spectroscopy) and found equivalent reliability between the three parameters in predicting DIND, favoring THRT due to the time consuming nature of the latter two [22].

Another interesting observation emerging from our small retrospective cohort study is that the systolic velocity in the MCA was elevated in five patients from their ictus; however, it did not translate into the development of DIND. We hypothesize that this could be a secondary response to either aggressive initial management or to the systemic stress response seen in SAH patients.

The analysis and comparison of the data obtained from THRT and radiological imaging measurements suggests that the latter methods are only moderately predictive of subsequent DIND development. We found that of all the CTP maps the MTT and TTP were the most predictive. These observations will have to be validated in a larger cohort studies.

This study suffers from the inherent drawbacks of any retrospective review. Our sample size is small but illustrative to this test. Longitudinal THRT testing was not done in all patients and would be considered in future projects.

Our results suggest the utility of THRT in detecting patients at increased risk of DIND from the time of admission, which if supported by prospective trials, will allow supporting a watchful clinical approach to their clinical course of investigation and management. It can also lead us to better select patients for trials involving early interventions in SAH and DIND, as it might serve as discriminatory test to stratify high versus low risk candidates. Understanding the trend of THRT during the period of DIND and its resolution might prove useful as well.

## Conclusions

Early abnormal THRT can predict future development DIND in patients with SAH, independent of flow velocities or the clinical grade of the SAH.

## References

[1] Modi NJ, Agrawal M, Sinha VD, D’Andrea A, Conte M, Cavallaro M (2016). Post-traumatic subarachnoid hemorrhage: a review. Neurol India.

[2] Perrein A, Petry L, Reis A, Baumann A, Mertes P, Audibert G (2015). Cerebral vasospasm after traumatic brain injury: an update. Minerva Anestesiol.

[3] Brown RJ, Kumar A, Dhar R, Sampson TR, Diringer MN (2013). The relationship between delayed infarcts and angiographic vasospasm after aneurysmal subarachnoid hemorrhage. Neurosurgery.

[4] Dorsch NW, King MT (1994). A review of cerebral vasospasm in aneurysmal subarachnoid haemorrhage part I: incidence and effects. J Clin Neurosci.

[5] Rowland MJ, Hadjipavlou G, Kelly M, Westbrook J, Pattinson KT (2012). Delayed cerebral ischaemia after subarachnoid haemorrhage: looking beyond vasospasm. Br J Anaesth.

[6] Mills JN, Mehta V, Russin J, Amar AP, Rajamohan A, Mack WJ (2013). Advanced imaging modalities in the detection of cerebral vasospasm. Neurol Res Int.

[7] Schatlo B, Pluta RM (2007). Clinical applications of transcranial Doppler sonography. Rev Recent Clin Trials.

[8] Purkayastha S, Sorond F (2012). Transcranial Doppler ultrasound: technique and application. Semin Neurol.

[9] Wintermark M, Reichhart M, Thiran JP, Maeder P, Chalaron M, Schnyder P (2002). Prognostic accuracy of cerebral blood flow measurement by perfusion computed tomography, at the time of emergency room admission, in acute stroke patients. Ann Neurol.

[10] Murphy BD, Fox AJ, Lee DH, Sahlas DJ, Black SE, Hogan MJ (2006). Identification of penumbra and infarct in acute ischemic stroke using computed tomography perfusion-derived blood flow and blood volume measurements. Stroke.

[11] Giller CA (1991). A bedside test for cerebral autoregulation using transcranial Doppler ultrasound. Acta Neurochir.

[12] Chalouhi N, Ali MS, Jabbour PM, Tjoumakaris SI, Gonzalez LF, Rosenwasser RH (2012). Biology of intracranial aneurysms: role of inflammation. J Cereb Blood Flow Metab.

[13] Cloft HJ, Joseph GJ, Dion JE (1999). Risk of cerebral angiography in patients with subarachnoid hemorrhage, cerebral aneurysm, and arteriovenous malformation: a meta-analysis. Stroke.

[14] Rathakrishnan R, Gotman J, Dubeau F, Angle M (2011). Using continuous electroencephalography in the management of delayed cerebral ischemia following subarachnoid hemorrhage. Neurocrit Care.

[15] Werner C, Engelhard K (2007). Pathophysiology of traumatic brain injury. Br J Anaesth.

[16] Budohoski KP, Reinhard M, Aries MJ, Czosnyka Z, Smielewski P, Pickard JD (2012). Monitoring cerebral autoregulation after head injury. Which component of transcranial Doppler flow velocity is optimal?. Neurocrit Care.

[17] Czosnyka M, Smielewski P, Piechnik S, Steiner LA, Pickard JD, Budohoski KP (2001). Cerebral autoregulation following head injury. J Neurosurg.

[18] Czosnyka M, Brady K, Reinhard M, Smielewski P, Steiner LA, Piechnik S (2009). Monitoring of cerebrovascular autoregulation: facts, myths, and missing links cerebral autoregulation following head injury. Neurocrit Care.

[19] Smielewski P, Czosnyka M, Kirkpatrick P, Pickard JD (1997). Evaluation of the transient hyperemic response test in head-injured patients. J Neurosurg.

[20] Lam JM, Smielewski P, Czosnyka M, Pickard JD, Kirkpatrick PJ (2000). Predicting delayed ischemic deficits after aneurysmal subarachnoid hemorrhage using a transient hyperemic response test of cerebral autoregulation. Neurosurgery.

[21] Lang EW, Mehdorn HM, Dorsch NW, Czosnyka M (2002). Continuous monitoring of cerebrovascular autoregulation: a validation study. J Neurol Neurosurg Psychiatry.

[22] Budohoski KP, Czosnyka M, Smielewski P, Varsos GV, Kasprowicz M, Brady KM (2013). Cerebral autoregulation after subarachnoid hemorrhage: comparison of three methods. J Cereb Blood Flow Metab.

